# High salt intake damages myocardial viability and induces cardiac remodeling *via* chronic inflammation in the elderly

**DOI:** 10.3389/fcvm.2022.952691

**Published:** 2022-10-06

**Authors:** Ke Li, Huajing Song, Fang Wei, Di Liu, Yingxin Zhao, Haipeng Yin, Yi Cui, Hua Zhang, Zhendong Liu

**Affiliations:** ^1^Department of Cardiology, Shandong Provincial Hospital Affiliated to Shandong First Medical University, Jinan, China; ^2^School of Clinical and Basic Medical Sciences, Shandong First Medical University, Shandong Academy of Medical Sciences, Jinan, China; ^3^Department of Cardiology, Jinan Central Hospital Affiliated to Shandong First Medical University, Jinan, China; ^4^Department of Radiology, Qilu Hospital of Shandong University, Jinan, China

**Keywords:** cardiac function, cardiac structure, inflammation, myocardial viability, salt intake

## Abstract

**Background:**

The heart is an important target organ for the harmful effects of high dietary salt intake. However, the effects and associations of high salt intake on myocardial viability, cardiac function changes, and myocardial remodeling are unclear.

**Methods:**

A total of 3,810 participants aged 60 years and older were eligible and enrolled from April 2008 to November 2010 and from August 2019 to November 2019 in the Shandong area of China. Salt intake was estimated using 24-h urine collection consecutively for 7 days. Myocardial strain rates, cardiac function and structure, and serum high-sensitivity C-reactive protein (hsCRP) levels were assessed. Participants were classified into low (*n* = 643), mild (*n* = 989), moderate (*n* = 1,245), and high (*n* = 933) groups, corresponding to < 6, 6–9, 9–12, and >12 g/day of salt intake, respectively, depending on the salt intake estimation.

**Results:**

The global early diastolic strain rate (SRe) and late diastolic strain rate (SRa) in the high group were 1.58 ± 0.26, 1.38 ± 0.24. respectively, and significantly lower compared with the low, mild, and moderate groups (all *P* < 0.05). The global systolic strain rate (SRs) in the high group was −1.24 ± 0.24, and it was higher than those in the low, mild, and moderate groups (all *P* < 0.05). Salt intake was independently and positively correlated with global SRs, Tei index, and the parameters of left ventricular structure separately; negatively correlated with global SRe and SRa, left ventricular short axis shortening rate, left ventricular ejection fraction after adjusting for confounders (all *P*_adjusted_ < 0.001). Hayes process analyses demonstrated that the mediating effects of hsCRP on global SRe, SRa, and SRs; Tei index; and left ventricular remodeling index were −0.013 (95% CI: −0.015 to −0.010), −0.010 (−0.012 to −0.008), 0.008 (0.006–0.010), 0.005 (0.003–0.006), and 0.010 (0.009–0.012), respectively (all *P*_adjusted_ < 0.001).

**Conclusion:**

Our data indicate that excess salt intake is independently associated with the impairment in myocardial viability and cardiac function, as well as myocardial remodeling. Chronic inflammation might play a mediating role in the association between high salt intake and cardiac function damage and myocardial remodeling.

## Introduction

Salt is necessary for the maintenance of normal physiological reactions in the body and evidence has shown that the average daily salt consumption is >10 g in most populations, which is double the recommendation by the World Health Organization (WHO) of 5 g/day, with the target for many countries being 6 g/day (1, 2). High salt intake is believed to lead to a significant increase in the damage to numerous organs such as the heart, blood vessels, kidneys, and brain (1–4) and an association between high salt intake and heart failure has been established (5–7). High salt intake can cause a sustained increase in the left ventricular load, which results in functional and structural changes in the left ventricle (8, 9).

The strain rate (SR) is a sensitive and direct index of myocardial viability and changes in cardiac function (10–13). SR quantifies the velocity of myocardial contraction and correlates with cardiomyocyte hypertrophy and fibrosis (14, 15). Potter and Marwick (16) found that the strain rate is an important marker of the impaired myocardial longitudinal systolic function in patients who suffered from heart failure with preserved ejection fraction and asymptomatic diastolic dysfunction. In recent years, speckle-tracking echocardiography, with its good reliability and reproducibility, has been developed for the analysis of SR to early detect the ventricular movement and its segmental alterations (17, 18). Ishizu et al. (19) found that changes in the longitudinal strain of the left ventricle were significantly earlier than damage to the ejection function in a heart failure model. Soepriatna et al. (17) observed obvious myocardial strain changes in a mouse model of early ventricular remodeling. However, the associations and mediators between high salt intake and the changes in ventricular SR and cardiac structure are still incompletely understood.

High salt intake has been demonstrated to promote inflammatory responses (20) which plays a prominent role in the development of cardiovascular disease. This increased systemic inflammation is consistent with the increased risk of cardiovascular events (21). High-sensitivity C-reactive protein (hsCRP) is a non-specific inflammatory marker (22). Increased hsCRP indicates a high level of systemic inflammatory response in the body and is independently related to the development of cardiovascular disease (23, 24). Thus, we hypothesized that the inflammatory response, which is represented by hsCRP in this study, might have an important mediating effect between high salt intake and early functional and structural changes in the left ventricle.

The main goal of this study was to investigate the correlations between high salt intake and functional and structural changes in the left ventricle, and to clarify the mediating role of hsCRP in these correlations.

## Methods

This study was approved by the Research Ethics committee of the Institute of Basic Medicine, Shandong Academy of Medical Sciences, Jinan, China, and was conducted in compliance with the Declaration of Helsinki. Written informed consent was obtained from each participant.

### Study population

This study was based on baseline data from two population-based and prospective cohort studies (study ID: ChiCTR1900023580 and ChiCTR-EOC-17013598). From April 2007 to November 2010 and from August 2019 to November 2019, 12,763 individuals aged 60 years or older, both male and female, were recruited from the Shandong area, China. Among them, 4,502 completed 24-h urine collection for 7 consecutive days, echocardiography, and blood sample collection. Finally, 3,810 eligible participants were included in this study. The exclusion criteria were: atrial fibrillation and various diagnosed arrhythmias, angina pectoris, myocardial infarction, congenital heart disease, cardiac insufficiency, primary cardiomyopathy, rheumatic heart disease, aortic valve stenosis and/or accompanied regurgitation, aortic valve surgery, other severe non-aortic valve regurgitation diseases, chronic and acute renal diseases, dialysis, severe liver diseases, malignancy, psychological diseases, dementia, connective tissue disease, acute or chronic infectious diseases, drug and alcohol abuse, and unwillingness to provide informed consent.

### Salt intake estimation

In this study, a 24-h urine collection within 7 consecutive days was used to estimate the salt intake. This is a widely used method for estimating salt intake and has been endorsed by the WHO (25, 26). The participants were asked to maintain a normal daily dietary pattern during the urine collection period. An electrolyte analyzer (DSI-905, Shanghai Xunda Medical Instrument Co., Ltd, Shanghai, China) was used to measure the urine Na^+^ concentration. The salt intake was calculated as the product of the Na^+^ concentration (mmol/L) and urine volume (L/day) divided by 17 (mmol/g). The quality and quantity of the urine sample were monitored using urine creatinine levels. Participants were grouped into low, mild, moderate, and high salt intake groups. Participants who consumed < 6 g/day of salt were classified into the low group in this study according to the dietary guidelines for Chinese residents published in 2016 (27). After that, the participants were grouped for every 3 g/day increment in salt intake. The high salt intake group was defined as >12 g/day of salt intake because fewer participants consumed more than 15 g/day of salt. In brief, participants were grouped into low, mild, moderate, and high salt intake, corresponding to < 6, 6–9, 9–12, and >12 g/day of salt intake, respectively.

### Echocardiography

Within the period of urine sample collection, ultrasonography was performed using a high-resolution ultrasound with a 2–5 MHz cardiac and 3D matrix transducer (Vivid 7, GE Medical Systems Ultrasound Israel Ltd, Israel) by experienced ultrasonographers who were blinded to the participants' salt intake and clinical details.

The left ventricular end-diastolic diameter, end-systolic diameter, interventricular septal thickness, posterior wall thickness, cardiac isovolumic contraction time, isovolumic relaxation time, and left ventricular ejection time were obtained using a 2D M-mode method at a speed of 100 ms. Then, left ventricular function, including the left ventricular short axis shortening rate (LVFS), left ventricular ejection fraction (LVEF, %), Tei index, left ventricular conformation including the left ventricular mass (LVM), left ventricular mass index (LVMI), and left ventricular remodeling index (LVRI) were calculated (28–30). The Tei index was described as an independent evaluation index of overall myocardial performance and calculated using the formula (isovolumic contraction time + isovolumic relaxation time)/ejection time (31). The normal value of the left ventricular Tei index was < 0.40 (32). After a deep breath, participants were asked to hold their breath to obtain the clear apical four-, three-, two-chamber and short-axis views (frame rates, 100–150 f/s) for three consecutive cycles using a tissue velocity imaging model and the images were stored for offline analyses.

### Echocardiographic image analysis

Speckle-tracking echocardiography with a 2D-STE software program (2D-Strain, EchoPAC PC113, GE Healthcare, USA) was used to analyze the apical four-, three-, and two-chamber views. Points were placed on the sub-endocardial myocardial layer of the apex, middle, and basal segments of the left ventricular posterior septum, lateral wall, anterior wall, inferior wall, anterior septum, and posterior wall. The software program automatically calculated the 2D global myocardial strain of the left ventricle. The global systolic SR (SRs), early diastolic SR (SRe), and late diastolic SR (SRa) were calculated from segmental averaging of the views at the apical, middle, and basal segments for further analyses (33).

All processing and analyses of echocardiographic data were conducted by experienced echocardiogram experts who were blinded to participants' dietary salt intake and clinical data. After independently testing 150 participants in random, the inter-observer variability was 0.93 (95% CI: 0.89–0.96) for global SRe, 0.95 (95% CI: 0.92–0.98) for global SRa, and 0.95 (95% CI: 0.93–0.98) for global SRs. The intra-observer variability was Echo PAC 0.90 (95% CI: 0.87–0.93) for global SRe, 0.93 (95% CI: 0.89–0.97) for global SRa, and 0.93 (95% CI: 0.90–0.96) for global SRs.

### hsCRP assessment and clinical laboratory measurements

An intravenous blood sample from each participant was obtained in the morning after overnight fasting. Serum and plasma were separated and frozen immediately, and stored at −80°C for further analysis. The serum levels of hsCRP were assessed using enzyme-linked immunosorbent assay kits (#PC198, Beyotime, Shanghai, China) following the manufacturer's instructions. A Hitachi 7600 automated biochemical analyzer (Hitachi, Ltd, Tokyo, Japan) was used to measure the plasma levels of total cholesterol (TCHO), triglycerides, high-density lipoprotein cholesterol (HDL-C), low-density lipoprotein cholesterol (LDL-C), and fasting plasma glucose (FPG) and each sample was tested in duplicate and the mean value used for further analyses.

### Covariates

Smoking was defined as having consumed more than 100 cigarettes in the past and current smoking (34). Drinking was defined as alcohol consumption in the past for more than 6 months and drinking at least once a week on average (34). Exercise was defined as engaging in physical exercise at least 3 times a week for more than 30 mins each time (34). Myocardial ischemia was diagnosed using an electrocardiogram with the ST segment lowered by at least 0.1 mV (35).

### Statistical analyses

Data were analyzed using SPSS for Windows (version 26.0; SPSS Inc., Chicago, IL, USA), and depending on the normality of the data distribution, continuous variables were expressed as the mean ± standard deviation (SD) or the median with interquartile range (IQR; the range between the 25th and 75th percentiles). Categorical variables were summarized as frequencies and percentages. The Kolmogorov-Smirnov test was used to detect the normality of continuous variable distribution. The differences in continuous variables between groups were assessed using the one-way analysis of variance with the Bonferroni *post hoc* test or Kruskal-Wallis tests depending on the normality of the data distribution. The chi-square test was used to determine the differences in categorical variables between groups. Pearson's or Spearman's correlation coefficients was used to assess correlations between continuous variables depending on the normality of the data distribution. A multiple linear regression analysis was used to identify factors possibly and independently associated with the level of hsCRP and the parameters of cardiac structure and function profile. The cut-off for the retention and elimination of variables was set to 0.05 in the model. SPSS Hayes process version 4.0 was used to examine the moderating and mediating effect of hsCRP in the process of high salt intake resulting in damage to cardiac structure and function. The dependent variables included myocardial strain rates and the parameters of left ventricular function and structure separately. The independent variables were the groups as classified by salt intake. The level of hsCRP was entered as a mediator. In the model, a *P-value* of the mediating effect of < 0.05 indicates a significant mediating effect. Model 1 was adjusted for age, sex, and body mass index. Model 2 was adjusted for the confounders in model 1 and smoking, drinking, exercise, heart rate, systolic and diastolic blood pressure, blood lipids, and FPG. Model 3 was adjusted for the confounders in model 2 and the medical history (of hypertension, diabetes, and dyslipidemia) and medications (anti-hypertensive, glucose-lowering, anti-dyslipidemia, and antiplatelet). A two-sided *P* < 0.05 was considered statistically significant.

## Results

### Demographic and clinical characteristics of the participants

**Figure 1:**
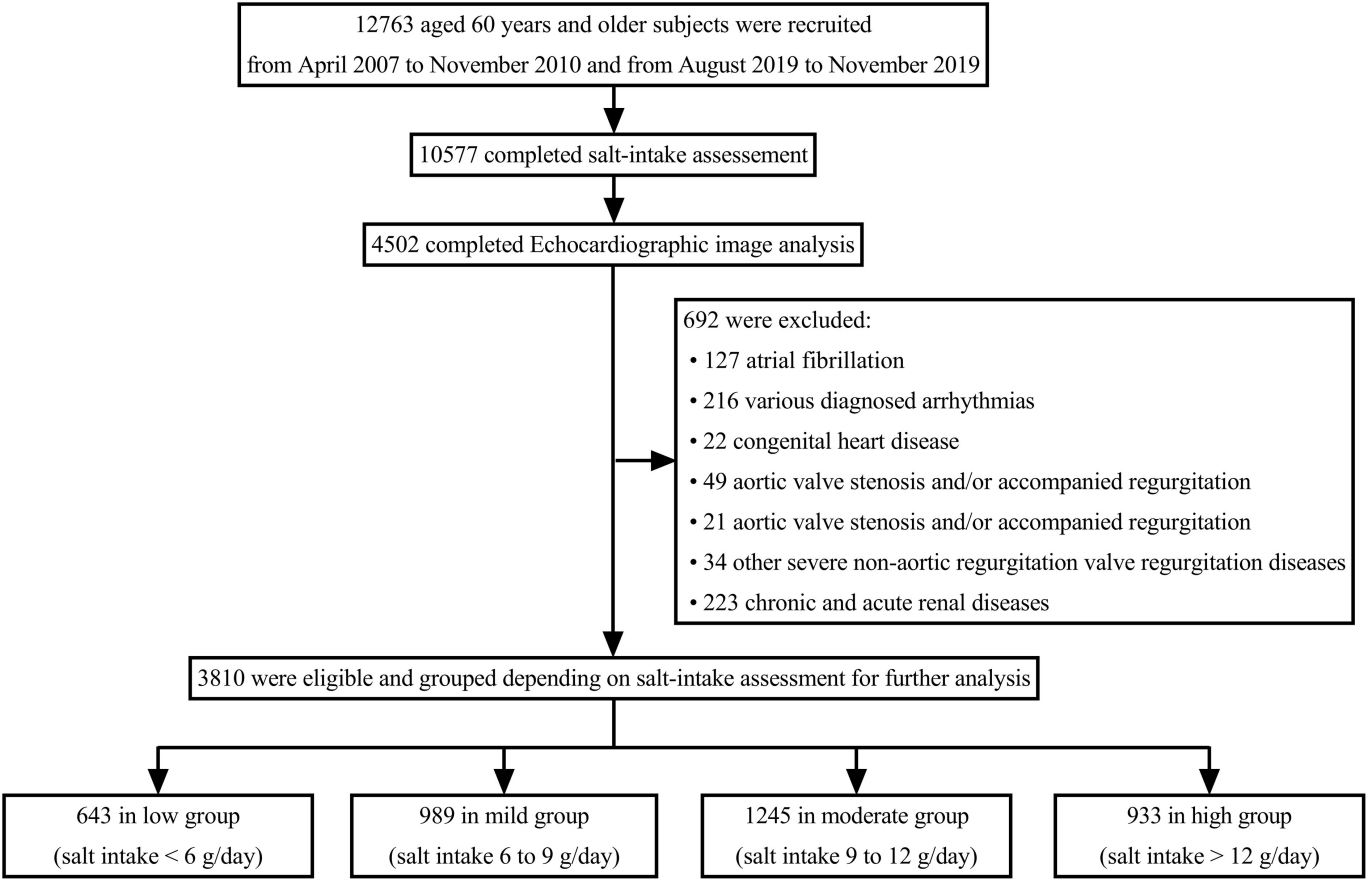
Flowchart of the participant recruitment process.

Figure 1 shows a flowchart of the patient recruitment procedure in this study. Among the 3,810 participants, 643 were classified into the low group, 989 into the mild group, 1,245 into the moderate group, and 933 into the high group, according to the salt intake estimation. Table 1 details the demographic and clinical characteristics of the four groups. Histories of hypertension in the mild, moderate, and high groups were higher than those in the low group (*P* < 0.05). Myocardial ischemia and systolic blood pressure were higher in the moderate and high groups than in the low group, and higher in the high group than in the mild group (*P* < 0.05). HDL-C was lower in the high group than in the low group (*P* < 0.05). Supplementary Table S1 presents the differences in the demographic and clinical characteristics of the participants between the two cohorts.

**Table 1:** Demographic and clinical characteristics of the study participants.

**Salt intake group**	** < 6 g/day**	**6–9 g/day**	**9–12 g/day**	**>12 g/day**	***P-value***
	**Low group** ** (*n* = 643)**	**Mild group** ** (*n* = 989)**	**Moderate group** ** (*n* = 1,245)**	**High group** ** (*n* = 933)**	
Age, years	68.89 ± 6.21	68.39 ± 5.80	68.46 ± 6.03	68.46 ± 6.07	0.381
Sex, female (%)	301 (46.8)	476 (48.1)	601 (48.2)	440 (47.1)	0.908
Smoking, *n* (%)	166 (25.8)	309 (31.2)	387 (31.0)	274 (29.3)	0.074
Drinking, *n* (%)	179 (27.8)	294 (29.7)	360 (28.9)	276 (29.5)	0.865
Exercise, *n* (%)	407 (63.2)	647 (65.4)	837 (67.2)	626 (67.0)	0.311
Myocardial ischemia, *n* (%)	181 (28.1)	297 (30.0)	413 (33.2)*	336 (36.0)*^†^	0.003
Hypertension, *n* (%)	400 (62.2)	715 (72.2)*	909 (73.0)*	685 (73.4)*	< 0.001
Anti-hypertensive medication, *n* (%)				
Diuretics, *n* (%)	182 (28.3)	288 (29.1)	389 (31.2)	262 (28.1)	0.358
Beta-blocker, *n* (%)	113 (17.6)	166 (16.8)	207 (16.6)	192 (20.6)	0.078
Calcium channel blocker, *n* (%)	147 (22.9)	271 (27.4)	302 (24.3)	210 (22.5)	0.058
Angiotensin-converting enzyme inhibitor, *n* (%)	176 (27.4)	268 (27.1)	328 (26.3)	224 (24.0)	0.362
Angiotensin receptor blocker, *n* (%)	122 (19.0)	183 (18.5)	227 (18.2)	202 (21.7)	0.198
Diabetes, *n* (%)	88 (13.6)	122 (12.3)	158 (12.6)	121 (12.9)	0.879
Lowering-glucose medication, *n* (%)	85 (13.2)	119 (12.0)	155 (12.4)	119 (12.7)	0.908
Dyslipidemia, *n* (%)	333 (51.7)	518 (52.3)	682 (54.7)	519 (55.6)	0.310
Anti-dyslipidemia medication, *n* (%)	111 (17.2)	167 (16.8)	199 (15.9)	161 (17.2)	0.842
Antiplatelet medication, *n* (%)	196 (30.4)	312 (31.5)	403 (32.3)	297 (31.8)	0.869
BMI, kg/m^2^	25.39 ± 3.24	25.64 ± 3.38	25.45 ± 3.48	25.44 ± 3.54	0.424
SBP, mmHg	144.95 ± 18.18	146.62 ± 18.69	147.38 ± 19.01*	148.79 ± 19.36*^†^	< 0.001
DBP, mmHg	71.04 ± 9.60	71.96 ± 9.65	71.32 ± 9.05	71.38 ± 9.41	0.218
Heart rate, beats/min	72.13 ± 9.03	72.44 ± 9.03	72.65 ± 9.27	72.74 ± 9.22	0.567
FPG, mmol/L	5.43 ± 1.57	5.52 ± 1.60	5.53 ± 1.67	5.58 ± 1.69	0.360
TCHO, mmol/L	4.78 ± 0.84	4.79 ± 0.88	4.78 ± 0.93	4.74 ± 0.86	0.627
TG, mmol/L	1.62 ± 0.61	1.62 ± 0.67	1.62 ± 0.68	1.64 ± 0.70	0.859
HDL-C, mmol/L	1.20 ± 0.42	1.15 ± 0.39	1.15 ± 0.41	1.13 ± 0.39*	0.016
LDL-C, mmol/L	2.84 ± 0.69	2.90 ± 0.77	2.89 ± 0.83	2.86 ± 0.81	0.449
hsCRP, mg/dL	0.37 (0.27 to 0.46)	0.51 (0.41 to 0.64)*	0.65 (0.51 to 0.80)*^†^	0.82 (0.69 to 0.93)*^†^^‡^	< 0.001

The results are presented as the mean ± SD or the median (IQR, the range from the 25th to 75th percentile), or as frequencies and percentages. BMI indicates body mass index; SBP, systolic blood pressure; DBP, diastolic blood pressure; FPG, fasting plasma glucose; TCHO, total cholesterol; TG, triglyceride; HDL-C, high-density lipoprotein cholesterol; LDL-C, low-density lipoprotein cholesterol; hsCRP, high-sensitivity C-reactive protein. ^*^P < 0.05, compared with low group;^†^P < 0.05, compared with the mild group;^‡^P < 0.05, compared with moderate group.

### Contribution of salt intake to myocardial strain rate

First, we compared the differences in the myocardial strain rates among the four groups (Table 2). The global SRe and SRa were significantly decreased while the global SRs increased from the low group to the mild, moderate, and high groups (all *P* < 0.05).

**Table 2:** Differences in the myocardial strain rates and left ventricular structure and function between the four groups.

**Salt intake group**	** < 6 g/day**	**6–9 g/day**	**9–12 g/day**	**> 12 g/day**	***P-value***
	**Low group** ** (*n* = 643)**	**Mild group** ** (*n* = 989)**	**Moderate group** ** (*n* = 1,245)**	**High group** ** (*n* = 933)**	
Global strain rates				
SRe	2.27 ± 0.25	2.02 ± 0.27*	1.81 ± 0.25*^†^	1.58 ± 0.26*^†^^‡^	< 0.001
SRa	1.99 ± 0.24	1.75 ± 0.26*	1.58 ± 0.23*^†^	1.38 ± 0.24*^†^^‡^	< 0.001
SRs	−1.80 ± 0.21	−1.57 ± 0.25*	−1.43 ± 0.25*^†^	−1.24 ± 0.24*^†^^‡^	< 0.001
Cardiac function index				
LVFS	44.72 ± 3.37	43.05 ± 3.43*	41.53 ± 3.47*^†^	40.69 ± 3.45*^†^^‡^	< 0.001
LVEF	67.69 ± 4.93	66.07 ± 5.67*	64.83 ± 5.17*^†^	61.14 ± 5.38*^†^^‡^	< 0.001
Tei index	0.36 (0.30–0.43)	0.43 (0.35–0.52)*	0.46 (0.38–0.58)*^†^	0.54 (0.44–0.66)*^†^^‡^	< 0.001
Ventricular remodeling index				
LVM	132.33 (116.54–148.41)	143.11 (128.50–162.81)*	156.17 (139.94–176.19)*^†^	183.41 (161.26–205.51)*^†^^‡^	< 0.001
LVMI	79.11 (69.03–90.01)	85.01 (74.29–98.18)*	95.00 (82.61–108.74)*^†^	111.09 (95.43–127.54)*^†^^‡^	< 0.001
LVRI	1.42 ± 0.19	1.48 ± 0.19*	1.51 ± 0.21*^†^	1.58 ± 0.18*^†^^‡^	< 0.001

The results are presented as the mean ± SD or the median (IQR, the range from the 25th to 75th percentile). SRe, early diastolic maximum strain rate; SRa, late diastolic maximum strain rate; SRs, systolic maximum strain rate; LVFS, left ventricular short axis shortening rate; LVEF, left ventricular ejection fraction; LVM, left ventricular mass; LVMI, left ventricular mass index; LVRI, left ventricular remodeling index. ^*^P < 0.05, compared with the low group;^†^P < 0.05, compared with the mild group;^‡^P < 0.05, compared with the moderate group.

Next, we analyzed the association between salt intake and myocardial strain rates (Table 3; Supplementary Table S2). The results showed that salt intake was significantly and negatively correlated with global SRe and SRa but positively correlated with global SRs (all *P* < 0.001, Supplementary Table S2). After adjustment for confounders (including a history of hypertension, systolic and diastolic blood pressure, and anti-hypertensive medication), salt intake was independently associated with global SRe, SRa, and SRs (all *P*_adjusted_ < 0.001, Table 3).

**Table 3:** Association between salt intake and strain rates and left ventricular function and structure.

	**Model 1**		**Model 2**		**Model 3**	
	**Beta (95% CI)**	***P-value***	**Beta (95% CI)**	***P-value***	**Beta (95% CI)**	***P-value***
Global strain rates						
SRe	−0.085 (−0.088 to −0.082)	< 0.001	−0.085 (−0.088 to −0.082)	< 0.001	−0.085 (−0.088 to −0.082)	< 0.001
SRa	−0.074 (−0.077 to −0.071)	< 0.001	−0.074 (−0.077 to −0.072)	< 0.001	−0.074 (−0.077 to −0.072)	< 0.001
SRs	0.068 (0.065–0.071)	< 0.001	0.068 (0.066 to 0.071)	< 0.001	0.069 (0.066–0.072)	< 0.001
Cardiac function index						
LVFS	−0.504 (−0.545 to −0.464)	< 0.001	−0.503 (−0.546 to −0.463)	< 0.001	−0.503 (−0.543 to −0.462)	< 0.001
LVEF	−0.742 (−0.806 to −0.678)	< 0.001	−0.738 (−0.801 to −0.675)	< 0.001	−0.737 (−0.801 to −0.673)	< 0.001
Tei index	0.021 (0.020 to 0.023)	< 0.001	0.021 (0.020 to 0.023)	< 0.001	0.021 (0.020 to 0.023)	< 0.001
Ventricular remodeling index						
LVM	6.013 (5.671 to 6.356)	< 0.001	5.988 (5.647 to 6.328)	< 0.001	5.985 (5.646 to 6.324)	< 0.001
LVMI	3.850 (3.625 to 4.074)	< 0.001	3.845 (3.619 to 4.071)	< 0.001	3.834 (3.610 to 4.059)	< 0.001
LVRI	0.019 (0.017 to 0.021)	< 0.001	0.019 (0.017 to 0.021)	< 0.001	0.019 (0.017 to 0.021)	< 0.001

Model 1 was adjusted for age, sex, and body mass index. Model 2 was adjusted for the confounders in model 1 and smoking, drinking, exercise, heart rate, systolic and diastolic blood pressure, blood lipids, and FPG. Model 3 was adjusted for the confounders in model 2 and the medical history (of hypertension, diabetes, and dyslipidemia) and medications (anti-hypertensive, glucose-lowering, anti-dyslipidemia, and antiplatelet). SRe indicates early diastolic maximum strain rate; SRa, late diastolic maximum strain rate; SRs, systolic maximum strain rate; LVFS, left ventricular short axis shortening rate; LVEF, left ventricular ejection fraction; LVM, left ventricular mass; LVMI, left ventricular mass index; LVRI, left ventricular remodeling index.

### Contribution of salt intake to cardiac function and myocardial remodeling

Table 2 presents the differences in left ventricular structure and function between the four groups. Tei index, LVM, LVMI, and LVRI were significantly increased, while LVFS and LVEF decreased from the low group to the mild, moderate, and high groups (all *P* < 0.05).

Salt intake was significantly and positively correlated with Tei index, LVM, LVMI, and LVRI, but negatively correlated with LVFS and LVEF (all *P* < 0.001, Supplementary Table S2). After adjustment for confounders, salt intake was independently associated with LVFS, LVEF, Tei index, LVM, LVMI, and LVRI (all *P*_adjusted_ < 0.001, Table 3).

### Correlation between salt intake and hsCRP

The serum level of hsCRP was significantly increased from the low group to the mild, moderate, and high groups (*P* < 0.05, Table 1). Salt intake was independently associated with the serum level of hsCRP after adjustment for confounders (beta-value: 0.041, 95% CI: 0.036–0.047; *P*_adjusted_ < 0.001).

### hsCRP correlated with myocardial strain rates and cardiac function and myocardial remodeling

The serum level of hsCRP was significantly and negatively correlated with global SRe and SRa, LVFS, and LVEF but positively correlated with global SRs, Tei index, LVM, LVMI, and LVRI (all *P* < 0.001, Supplementary Table S3). After adjustment for confounders, the serum level of hsCRP was still independently associated with global SRe, global SRa, global SRs, LVFS, LVEF, Tei index, LVM, LVMI, and LVRI (all *P*_adjusted_ < 0.001, Table 4).

**Table 4:** Association of hsCRP with myocardial strain rates and left ventricular structure and function.

	**Model 1**		**Model 2**		**Model 3**	
	**Beta (95% CI)**	***P-value***	**Beta (95% CI)**	***P-value***	**Beta (95% CI)**	***P-value***
Global strain rates						
SRe	−0.743 (−0.783 to −0.702)	< 0.001	−0.739 (−0.780 to −0.699)	< 0.001	−0.738 (−0.778 to −0.697)	< 0.001
SRa	−0.642 (−0.679 to −0.604)	< 0.001	−0.639 (−0.677 to −0.602)	< 0.001	−0.637 (−0.675 to −0.600)	< 0.001
SRs	0.570 (0.533 to 0.607)	< 0.001	0.570 (0.533–0.607)	< 0.001	0.571 (0.534–0.609)	< 0.001
Cardiac function index						
LVFS	−4.313 (−4.796 to −3.831)	< 0.001	−4.278 (−4.762 to −3.794)	< 0.001	−4.279 (−4.765 to −3.793)	< 0.001
LVEF	−6.420 (−7.177 to −5.663)	< 0.001	−6.406 (−7.161 to −5.652)	< 0.001	−6.371 (−7.130 to −5.612)	< 0.001
Tei index	0.208 (0.188 to 0.228)	< 0.001	0.206 (0.187 to 0.226)	< 0.001	0.206 (0.187 to 0.226)	< 0.001
Ventricular remodeling index						
LVM	65.053 (61.070 to 69.036)	< 0.001	64.997 (60.989 to 69.005)	< 0.001	64.943 (60.950 to 68.936)	< 0.001
LVMI	41.529 (38.892 to 44.166)	< 0.001	41.341 (38.693 to 43.988)	< 0.001	41.324 (38.686 to 43.963)	< 0.001
LVRI	0.260 (0.234 to 0.286)	< 0.001	0.260 (0.233 to 0.286)	< 0.001	0.260 (0.234 to 0.286)	< 0.001

Model 1 was adjusted for age, sex, and body mass index Model 2 was adjusted for the confounders in model 1 and smoking, drinking, exercise, heart rate, systolic and diastolic blood pressure, blood lipids, and FPG. Model 3 was adjusted for the confounders in model 2 and the medical history (of hypertension, diabetes, and dyslipidemia) and medications (anti-hypertensive, glucose-lowering, anti-dyslipidemia, and antiplatelet). SRe, early diastolic maximum strain rate; SRa, late diastolic maximum strain rate; SRs, systolic maximum strain rate; LVFS, left ventricular short axis shortening rate; LVEF, left ventricular ejection fraction; LVM, left ventricular mass; LVMI, left ventricular mass index; LVRI, left ventricular remodeling index.

### Mediating effect of hsCRP on the associations between salt intake and myocardial strain rates and cardiac function and myocardial remodeling

Through the Hayes process models, the results demonstrated that hsCRP plays a partially and significantly mediating effect on the association of salt intake with global SRe, global SRa, global SRs, LVFS, LVEF, Tei index, LVM, LVMI, and LVRI after adjustment for confounders (all *P*_adjusted_ < 0.001). The results are detailed in Table 5.

**Table 5:** The mediating effect of hsCRP in the association between salt intake and myocardial strain rates and left ventricular structure and function.

	**Model 1**		**Model 2**		**Model 3**	
	**Mediating effect (95% CI)**	***P-value***	**Mediating effect (95% CI)**	***P-value***	**Mediating effect (95% CI)**	***P-value***
Global strain rates						
SRe	−0.013 (−0.015 to −0.010)	< 0.001	−0.013 (−0.015 to −0.010)	< 0.001	−0.013 (−0.015 to −0.010)	< 0.001
SRa	−0.010 (−0.013 to −0.008)	< 0.001	−0.010 (−0.012 to −0.008)	< 0.001	−0.010 (−0.012 to −0.008)	< 0.001
SRs	0.008 (0.006 to 0.010)	< 0.001	0.008 (0.006 to 0.010)	< 0.001	0.008 (0.006 to 0.010)	< 0.001
Cardiac function index						
LVFS	−0.067 (−0.098 to −0.036)	< 0.001	−0.066 (−0.097 to −0.034)	< 0.001	−0.066 (−0.098 to −0.035)	< 0.001
LVEF	−0.105 (−0.155 to −0.056)	< 0.001	−0.106 (−0.155 to −0.057)	< 0.001	−0.106 (−0.156 to −0.057)	< 0.001
Tei index	0.005 (0.003 to 0.006)	< 0.001	0.005 (0.003 to 0.006)	< 0.001	0.005 (0.003 to 0.006)	< 0.001
Ventricular remodeling index						
LVM	1.914 (1.647 to 2.189)	< 0.001	1.91 (1.652 to 2.193)	< 0.001	1.911 (1.641 to 2.189)	< 0.001
LVMI	1.206 (1.032 to 1.387)	< 0.001	1.204 (1.034 to 1.374)	< 0.001	1.204 (1.034 to 1.379)	< 0.001
LVRI	0.010 (0.009 to 0.012)	< 0.001	0.010 (0.009 to 0.012)	< 0.001	0.010 (0.009 to 0.012)	< 0.001

Model 1 was adjusted for age, sex, and body mass index. Model 2 was adjusted for the confounders in model 1 and smoking, drinking, exercise, heart rate, systolic and diastolic blood pressure, blood lipids, and FPG. Model 3 was adjusted for the confounders in model 2 and the medical history (of hypertension, diabetes, and dyslipidemia) and medications (anti-hypertensive, glucose-lowering, anti-dyslipidemia, and antiplatelet). SRe, early diastolic maximum strain rate; SRa, late diastolic maximum strain rate; SRs, systolic maximum strain rate; LVFS, left ventricular short axis shortening rate; LVEF, left ventricular ejection fraction; LVM, left ventricular mass; LVMI, left ventricular mass index; LVRI, left ventricular remodeling index.

## Discussion

The main findings of this study were: (1) high salt intake was independently correlated with the changes in myocardial strain rates, and (2) hsCRP played a mediating role in the association between high salt intake and changing myocardial strain rates and left ventricular function and cardiac remodeling. Other important findings showed that a high salt intake was correlated with the changes in left ventricular function and myocardial remodeling and with high levels of hsCRP.

Evidence has shown that high salt intake is one important contributor to left ventricular dysfunction and myocardial remodeling (1–4). Our findings are consistent with the existing evidence (1–4). Salt intake was positively and independently correlated with hallmarks of left ventricular structure and function such as LVM, LVMI, LVRI, and Tei index, and negatively and independently correlated with LVFS and LVEF; however, it was impossible to clarify the causal relationship due to the inherent characteristics of a cross-sectional design.

An important finding of this study was the correlation between salt intake and myocardial strain rates. The myocardial strain rate has been demonstrated to be a direct and sensitive evaluation index for myocardial viability and early changes in cardiac function (10–13). In this study, we found significant differences in global SRe, SRa, and SRs between individuals with higher salt intake and those with lower salt intake. Salt intake was independently correlated with global SRe, SRa, and SRs. Our findings indicate that the early harmful cardiac effect of high salt intake might begin with myocardial viability and present as changes in the strain rate. Selvaraj et al. (36) reported that excessive salt intake is associated with a worsening of systolic myocardial strain and adverse myocardial remodeling. However, some studies such as that by Mak et al. (37) did not find an effect of subacute salt loading on myocardial strain rates in healthy young normotensive individuals. The disagreement between the findings of Mak's study and those of our study may be due to differences in participants' ages, sample sizes, and, most importantly, the duration of salt loading. The salt intake was estimated within 7 consecutive days under a daily dietary pattern in this study. This estimation method properly evaluates the long-term salt loading of the participants (38), and evidence has demonstrated that long-term salt loading activates chronic inflammatory responses (20, 39).

Excessive salt intake has been found to induce differentiation of CD4^+^ immune T cells into pro-inflammatory phenotype T helper cell 17 and upregulates pro-inflammatory genes expression by reducing intestinal Lactobacillus levels (39). Non-osmotic Na^+^ accumulation resulted by Na^+^ loading has been demonstrated to lead to a dysfunction of immune cells including macrophages in skin and muscle (40). High salt diet increases the expression levels of IL-1β, IL-6, and TNF-α in the mice cortex (41). In addition, high salt intake could damage redox systems to activate inflammatory response and impair the immune system (42). In our study, as expected, the level of hsCRP was found to increase along with the increase in salt intake. Thus, the data demonstrates that high salt dietary is an important contributor to inflammatory response.

Furthermore, hsCRP was significantly correlated with myocardial strain rates and the changes in left ventricular function and structure. Given that hsCRP is a non-specific chronic inflammatory marker and that an increase in the level hsCRP indicates the initiation of an inflammatory reaction and is related to the development of cardiovascular diseases (23, 24), these findings indicate that chronic inflammation may be an important mediator in the associations between excessive salt intake and changes in myocardial viability and cardiac function and myocardial remodeling. Thus, we performed Hayes process analyses to clarify the mediating role of hsCRP in the association between salt intake and the changes in myocardial viability and cardiac function and structure. Interestingly, the results confirmed our hypotheses. This indicates the measurement of anti-inflammatory marker levels could be a useful strategy in preventing harm from excessive salt intake (which leads to changes in cardiac function and myocardial remodeling) as well as lowering salt intake.

The strength of this study was that we explored the mediating effect of hsCRP in the association between salt intake and myocardial function impairment and cardiac remodeling. It provides an insight into the associations of excessive salt intake with changes in cardiac function and myocardial remodeling. In addition, we used a gold-standard measurement tool to estimate the daily salt intake under a daily dietary pattern. This minimized the bias in the salt intake estimation bias, because salt intake is significantly affected by dietary patterns (38). We also included blood pressure, heart rate, myocardial ischemia, hypertensive history, and anti-hypertensive medications as confounders in the analysis models used in this study. High salt intake is an important contributor to hypertension, and hypertension is associated with cardiac dysfunction and myocardial remodeling (43, 44). In addition, myocardial ischemia and different anti-hypertensive medications are regarded as important contributors to the changes in cardiac function and structure (45, 46).

However, some limitations must be considered. First, the daily dietary patterns of the participants were not fully considered. High dietary energy intake and low potassium intake have been demonstrated to be associated with cardiac dysfunction and cardiac remodeling (4, 47). Second, this study had a cross-sectional design, which did not clarify causality but only detected associations between study variables; therefore, prospective studies are needed. Third, the participants of the study were primarily enrolled from the Shandong area of China, which may have caused bias in the results.

In conclusion, our findings indicate that excessive salt intake is independently associated with the damage in myocardial viability and cardiac function, as well as adverse cardiac remodeling. A chronic inflammatory response might be an important mediator in the association between excessive salt intake and cardiac function damage and cardiac remodeling. More studies with large sample sizes and prospective designs involving multi-ethnic and multi-race individuals with full dietary patterns are needed in the future.

## Data availability statement

The raw data supporting the conclusions of this article will be made available by the authors, without undue reservation.

## Ethics statement

The studies involving human participants were reviewed and approved by Institute of Basic Medicine, Shandong Academy of Medical Sciences, Jinan, China. The patients/participants provided their written informed consent to participate in this study.

## Author contributions

KL and HS performed the analyses and wrote the methods, results, and discussion sections. FW developed the introduction section and collected the data. DL revised earlier versions of the manuscript. YZ co-performed the analyses, wrote the results, and revised earlier versions of the manuscript. HY and YC co-designed the study and developed the discussion sections. ZL and HZ designed and initiated the study, conducted the analyses, developed the introduction and discussion sections, and revised the final versions of the manuscript. All authors contributed significantly to the article and approved its submitted version.

## Funding

This work was supported by the National Natural Science Foundation of China (Grant Nos. 81973139 and 81670432), the Shandong Provincial Natural Science Foundation of China (Grant No. ZR2020MH043), the Medical and Health Science and Technology Development Plan Project of Shandong, China (Grant No. 202103010686), the Innovation Project of Shandong Academy of Medical Sciences, and the Academic Promotion Program of Shandong First Medical University.

## Conflict of interest

The authors declare that the research was conducted in the absence of any commercial or financial relationships that could be construed as a potential conflict of interest.

## Publisher's note

All claims expressed in this article are solely those of the authors and do not necessarily represent those of their affiliated organizations, or those of the publisher, the editors and the reviewers. Any product that may be evaluated in this article, or claim that may be made by its manufacturer, is not guaranteed or endorsed by the publisher.
