# Supplementary material for: High salt intake damages myocardial viability and induces cardiac remodeling via chronic inflammation in the elderly
Source: Front Cardiovasc Med. 2022 Oct 6;9:952691. doi: 10.3389/fcvm.2022.952691 (PMC9582749; doi:10.3389/fcvm.2022.952691)
Supplement: Supplementary file 1 [file Data_Sheet_1.pdf]

Supplementary Materials

Supplementary Table S1. Demographic and clinical characteristics of the study participants in the two cohorts

Cohort	April 2007 to November 2010 ChiCTR-EOC-17013598 (*n* = 3287)	August 2019 to November 2019 ChiCTR1900023580 (*n* = 523)	*P-value*
Age, years	68.09 ± 6.08	68.04 ± 5.41	0.859
Sex, female (%)	1578 (48.0)	240 (45.9)	0.368
Smoking, *n* (%)	984 (29.9)	152 (29.1)	0.685
Drinking, *n* (%)	942 (28.7)	167 (31.9)	0.126
Exercise, *n* (%)	2155 (65.6)	362 (69.2)	0.101
Myocardial ischemia, *n* (%)	1054 (32.1)	173 (33.1)	0.645
Hypertension, *n* (%)	2334 (71.0)	375 (71.7)	0.745
Anti-hypertensive medications			

Diuretics, *n* (%)	991 (30.1)	130 (24.9)	0.014
Beta-blocker, *n* (%)	574 (17.5)	104 (19.9)	0.178
Calcium channel blocker, *n* (%)	795 (24.2)	135 (25.8)	0.421
Angiotensin-converting enzyme inhibitor, *n* (%)	848 (25.8)	148 (28.3)	0.227
Angiotensin receptor blocker, *n* (%)	622 (18.9)	122 (23.3)	0.018
Diabetes, *n* (%)	422 (12.8)	67 (12.8)	0.986
Glucose-lowering medication, *n* (%)	411 (12.5)	67 (12.8)	0.844
Dyslipidemia, *n* (%)	1751 (53.3)	301 (57.6)	0.068
Anti-dyslipidemia medication, *n* (%)	489 (14.9)	149 (28.5)	<0.001
Antiplatelet medication, *n* (%)	1040 (31.6)	168 (32.1)	0.826
BMI, kg/m^2	25.45 ± 3.54	25.70 ± 3.37	0.131
SBP, mmHg	147.28 ± 19.44	147.34 ± 15.72	0.946
DBP, mmHg	71.51 ± 9.36	71.11 ± 8.17	0.356

Heart rate, beats/min	72.31 ± 9.21	72.02 ± 8.15	0.497
FPG, mmol/L	5.82 ± 1.69	5.79 ± 1.27	0.697
TCHO, mmol/L	4.77 ± 0.91	4.74 ± 0.68	0.470
TG, mmol/L	1.62 ± 0.70	1.63 ± 0.49	0.054
HDL-C, mmol/L	1.15 ± 0.41	1.17 ± 0.33	0.288
LDL-C, mmol/L	2.88 ± 0.81	2.86 ± 0.61	0.595
hsCRP, mg/dL	0.61 ± 0.23	0.62 ± 0.24	0.359

The results are presented as the mean ± SD or the median (IQR, the range from the 25th to 75th percentile), or as frequencies and percentages. BMI indicates body mass index; SBP, systolic blood pressure; DBP, diastolic blood pressure; FPG, fasting plasma glucose; TCHO, total cholesterol; TG, triglyceride; HDL-C, high-density lipoprotein cholesterol; LDL-C, low-density lipoprotein cholesterol; hsCRP, high-sensitivity C-reactive protein.

Supplementary Table S2. The correlations of salt intake with myocardial strain rates and left ventricular function and structure

Global strain rates			Left ventricular dysfunction and remodeling		
	Correlation coefficient	*P-value*		Correlation coefficient	*P-value*
SRe	-0.666	< 0.001	LVFS	-0.368	< 0.001
SRa	-0.642	< 0.001	LVEF	-0.347	< 0.001
SRs	0.607	< 0.001	Tei index	0.394	< 0.001
			LVM	0.515	< 0.001
			LVMI	0.481	< 0.001
			LVRI	0.253	< 0.001

The values present the Pearson or Spearman correlation coefficient depending on the normality of the data distribution for each variable. SRe, early diastolic maximum strain rate; SRa, late diastolic maximum strain rate; SRs, systolic maximum strain rate; LVFS, left ventricular short axis shortening rate; LVEF, left ventricular ejection fraction; LVM, left ventricular mass; LVMI, left ventricular mass index; LVRI, left ventricular remodeling index.

Supplementary Table S3. The correlations of hsCRP serum level with myocardial strain rates and left ventricular function and structure

Global strain rates			Left ventricular dysfunction and remodeling		
	Correlation coefficient	*P-value*		Correlation coefficient	*P-value*
SRe	-0.506	< 0.001	LVFS	-0.272	< 0.001
SRa	-0.480	< 0.001	LVEF	-0.260	< 0.001
SRs	0.440	< 0.001	Tei index	0.313	< 0.001
			LVM	0.452	< 0.001
			LVMI	0.414	< 0.001
			LVRI	0.300	< 0.001

The values present the Pearson or Spearman correlation coefficient depending on the normality of the variables. SRe, early diastolic maximum strain rate; SRa, late diastolic maximum strain rate; SRs, systolic maximum strain rate; LVFS, left ventricular short axis shortening rate; LVEF, left ventricular ejection fraction; LVM, left ventricular mass; LVMI, left ventricular mass index; LVRI, left ventricular remodeling index.
